# Effect of Total Flavones from Cuscuta Chinensis on Anti-Abortion via the MAPK Signaling Pathway

**DOI:** 10.1155/2018/6356190

**Published:** 2018-10-02

**Authors:** Hai-wang Wu, Yi-hui Feng, Dong-ying Wang, Wei-yu Qiu, Qing-ying Yu, Li-lin Yang, Chun Liang, Song-ping Luo, Jie Gao

**Affiliations:** ^1^Guangzhou University of Chinese Medicine, Guangzhou, China; ^2^Division of Life Science, Center for Cancer Research and State Key Lab for Molecular Neural Science, Hong Kong University of Science and Technology, Hong Kong, China; ^3^Department of Gynecology, The First Affiliated Hospital of Guangzhou University of Chinese Medicine, Guangzhou, China

## Abstract

For centuries, the Chinese herb Cuscuta chinensis has been applied clinically for abortion prevention in traditional Chinese medicine (TCM). Total flavones extracted from Cuscuta chinensis (TFCC) are one of the active components in the herb and also display anti-abortion effect similar to the unprocessed material. However, how TFCC exerts the anti-abortion effect remains largely unknown. In this study, we aim at characterizing the anti-abortion effects of TFCC and its underlying molecular mechanism in vitro and in vivo using human primary decidua cells and a mifepristone-induced abortion model in rat, respectively. The damage to the decidua caused by mifepristone in vivo was reversed by TFCC treatment in a dosage-dependent manner. High dosage of TFCC significantly upregulated the expression of estrogen receptor (ER), progesterone receptor (PR), and prolactin receptor (PRLR) in decidua tissue but downregulated the expression of p-ERK. Furthermore, we detected higher level of p-ERK and p-p38 in primary decidua cells from spontaneous abortion while treatment by TFCC downregulated their expression. Our results suggest TFCC mediates its anti-abortion effect by interfering with MAPK signaling pathway.

## 1. Introduction

*Cuscutae Semen* (Tu-Si-Zi) is the dried seed of* Cuscuta chinensis* Lam. or* cuta australis* R. Br. [1]. It has demonstrated therapeutic effect in various diseases, including neural disease [2], inflammation [3, 4], and cancer [5, 6]. Studies also showed that* Cuscutae Semen *has an anti-aging effect [7] and facilitates osteoporosis [8]. Furthermore, dated back as early as 2000 years ago, it has been described as one of the best Chinese medical plants recorded in ShenNong's Herbal and has been employed to treat spontaneous abortion by TCM practitioners due to its regulatory effect on ovulation and hormone regulation [9, 10]. A cohort study based on Taiwan population revealed that 96.17% (8766/8430) infertile women had sought TCM treatment and the most commonly prescribed herb was* Cuscutae Semen* [11].

In spite of its wide usage in Chinese population, C. chinensis is often prescribed and processed together with other herbal ingredients, and this becomes a barrier for identifying the effective components in the herb. Many studies have been devoted to isolate the active components in C. chinensis in order to expand its usage. The chemical components from C. chinensis consist of mainly flavonoids, steroids, volatile constituents, lignans, alkaloids, and polysaccharides. The flavonoids account for about 3% of the total chemical components [12] and are the major active ingredients in C. chinensis. Depending on the species, hosts, and different processing protocols, the exact compositions of TFCC might differ, but hyperoside, rutin, quercitrin, and quercetin remain the staple bioactive components of C. chinensis. Additionally, early studies found that TFCC regulates the endocrine-immune network in maternal-fetal interface and thereby reducing the abortion rate of the bromocriptine-stimulated abortion model in rat [13, 14]. Yet the exact biological function and molecular mechanism of TFCC at the maternal-fetal interface in human first-trimester pregnancy await further exploration. Mifepristone (RU486) is the progesterone antagonist and widely used for medical abortion. Thus, RU486 can be used to induce the abortion model. As one of the symptoms of abortion, uterine bleeding also can be observed in RU486-induced abortion mice and the volume of uterine bleeding is closely related to the Th1/Th2/Th17/Treg paradigm, which can be regulated by RU486 [15]. Recent studies revealed that RU486 can upregulate Monocyte Chemotactic Protein-3 (MCP3) at the implantation period caused immune abortion [16].

MAPK signaling pathway is involved in a series of physiological and pathological processes, including cell growth, development, differentiation, and apoptosis, all of which are essential to the invasion and proliferation of decidua stromal cells (DSCs) and trophoblast cells. Recent studies show that MAPK/p38 pathway, activated by upregulation of nucleotide-binding oligomerization domain containing 1 (NOD1) and nucleotide-binding oligomerization domain containing 2 (NOD2), inhibit the invasion of trophoblast cells [17]. Meanwhile, upregulation of S100P also activates the MAPK/p38 pathway, resulting in enhanced trophoblast-like cell proliferation [18]. Moreover, NF-*κ*B and ERK1/2 signaling pathway activated by IL-33 promotes proliferation and invasion by DSCs [19]. Additionally, IL-25 activates JNK and AKT signaling pathways, also leading to proliferation of DSCs [20].

Given the proliferative effect of MAPK on DSC, we decided to explore the role of MAPK signaling pathway in abortion prevention induced by TFCC. A previous study indicated that TFCC enhances the proliferation of the first trimester human trophoblast cells via ERK1/2 signaling pathway [21]. However, the exact role of MAPK signaling pathway on DSCs in preventing abortion by TFCC remains unclear. In this study, we investigated key molecules involved in MAPK signaling pathway in patients with spontaneous abortion and compared with the DSCs in patients with normal pregnancy. We hypothesize that MAPK signaling pathway may be among the causes of abortion and decidua dysfunction and TFCC may prevent abortion by inhibiting the MAPK signaling pathway.

## 2. Materials and Methods

### 2.1. Animals Study

The protocols for the animal studies were approved by institutional ethic committee and the studies were conducted in Guangdong Medical Lab Animal Center (No. B201411-6). Female Sprague-Dawley rats (269.55 ± 22.35 g) and male Sprague-Dawley rats (428.15 ± 22.55 g) were purchased from Guangdong Medical Lab Animal Center.

After one week of observation, female and male rats in mating trails were housed in groups at the ratio of 2:1 for 12 hours. Vaginal smears were taken every morning after mating. Observation of sperm and vaginal plug were considered as first day of pregnancy. Pregnant rats were randomized to five groups (10 rats each group): control group (Normal, Nor), model group (Model, Mod), dydrogesterone group (Positive, Pos), TFCC low dose group (TFCC1), and TFCC high dose group (TFCC2).

The pregnant rats in Nor Group were treated with distilled water by gavage. TFCC1 and TFCC2 groups were treated TFCC by gavage daily at a dose of 9.45 mg/ml and 18.9 mg/ml for ten days. Pos group received 0.604 mg/mL dydrogesterone (Abbott Biologicals B.V) for equivalent duration, 5 ml/kg once a day. On day 10 of pregnancy, rats in Pos Group were treated RU486 at the dose of 45 mg/kg (Hubei Gedian Humanwell Pharmaceutical) by gavage with gastric volume of 5 ml/kg. Nor group was given equivalent volume of normal saline (NS) in the same manner. SD rats were sacrificed under anesthesia 24 hours after administration of RU486. The third left embryo of pregnant rat was observed under the stereomicroscope.

### 2.2. Cells, Reagents, and Antibodies

Isolation, culture, and identification of primary decidual cells were performed according to protocols published in previous studies [22]. All patients gave their written informed consent according to Declaration of Helsinki, and we obtained the permission of Ethics Committee of The First Affiliated Hospital of Guangzhou University of Traditional Chinese Medicine (No. 2014050). DMEM/F12 with and without phenol red, FBS, and 0.25% trypsin were purchased from Gibco. Charcoal dextran-treated FBS were purchased from BI. Antibodies against MAPK signaling pathways were purchased from Cell Signaling Technology (#9926, #9910). Rabbit monoclonal anti-estrogen receptor alpha antibody [SP1], anti-GAPDH antibody [EPR16891], and rabbit polyclonal anti-progesterone receptor antibody, anti-FSH receptor antibody were products of Abcam as well as mouse monoclonal anti-prolactin receptor antibody [U5]. Mouse polyclonal anti-tubulin antibody was purchased from Beyotime (Jiangsu, China). Anti-Rabbit IgG (H + L), HRP Conjugate, and Anti-Mouse IgG (H + L), HRP Conjugate were purchased from Promega.

### 2.3. Herbal Extract Preparation

Total flavones were extracted from the dry seed of C. chinensis Lam. The quality control of TFCC was assessed by HPLC with Thermo Ultimate 3000 with Thermo Ultimate 3000 VWD-3x00 detector, Ecosil C18 pillar (4.6 mm × 250 mm, 5 *μ*m, Lubex, Japan).

The sample for animals and cell culture was prepared by extracting the powder with eight volumes of 95% of alcohol twice for 1hr each. The combined extracts (TFCC) were concentrated in vacuum and dissolved in pure water (19.45 mg/ml for TFCC1; 18.9 mg/mL for TFCC2) or in DMSO (10 mg/ml).

The mobile phase consisted of A (acetonitrile) and B (50 mM KH_2_PO_4_-H_3_PO_4_, pH 3.0) with a gradient elution flow rate of 1.0 ml/min. The detector wavelength was set at 220 nm. The gradient program (A/B, v/v) was as follows: 0-65 min, 10% → 28% A; 65-85 min: 28% → 52% A. The column temperature was set to 30°C. The HPLC chromatogram is shown in Figure 4. The contents of Rutin, Hyperoside, Quercitrin, and Quercetin in TFCC ethanol extracts were determined.

### 2.4. Analysis of Serum Sex Hormones

SD rats were euthanized under anesthesia 24 hours after administration of RU486. Abdominal aorta blood was extracted and centrifuged at 3000 rpm for 15 minutes. The serums were used for ELISA to detect FSH, LH, E2, P, and PRL according to the manufacturer's instructions (Bogoo Company Lit., Shanghai, China).

### 2.5. Quantitative PCR

Complementary strand DNA was synthesized from total RNA using the RT reagent Kit with gDNA Eraser (Takara, DRR047A) followed by qPCR using the SYBR® Premix Ex Taq™ (Tli RNaseH Plus) (Takara, DRR820A) on Bio-Rad IQ5 Real-Time PCR System (Bio-Rad, USA). Primers sequences were shown in Table 1. The reaction setup was as follows: 95°C for 30s followed by 40 cycles of 95°C for 5s and 60°C for 30s. *β*-Actin was used as an internal control to normalize the variability in expression levels.

### 2.6. Histopathology

After fixation of samples in 4% paraformaldehyde for 48h, the decidual tissues were dehydrated with ethanol and xylene, embedded in paraffin according to standard procedures and cut into serial sections of 4 *μ*m thickness. After deparaffinization, slides were stained with hematoxylin and eosin for routine histological examination. Images were captured by a digital video camera mounted on a light microscope.

### 2.7. Immunohistochemistry

The paraffin section of decidual tissue was cut into sections of 4*μ*m thickness and deparaffinized in xylene. After antigen retrieval for 15 minutes by microwave, the samples were incubated in primary antibody at 1:1000 at 4°C overnight. The slides were rinsed with PBS three times next day and incubated with secondary antibody Goat Anti-Rabbit lgG (HRP) (ab136817 1: 1000) at 37°C for 30 min according to the manufacturer's instructions. The slides were treated with DAB substrate for color development and then counterstained by hematoxylin. The stained slides were then dehydrated and mounted on slides for observation.

### 2.8. Western Blotting

The samples were mixed with 5×Loading buffer and heated for 5 min for protein denaturation. The samples were then stored at -20°C after centrifugation and proceed to the next step. Samples were separated by SDS–PAGE at 70 voltages for 30 minutes and then the voltage was adjusted to 120V for 1h. Afterwards, the samples were transferred to PVDF membrane with 220mA for 1h. The membranes were blocked with 5% BSA or nonfat dried milk diluted in TBS containing 0.1% Tween 20 or for 1h at room temperature (RT) and then rinsed with TBS-T for three times. The membranes were incubated with appropriately diluted primary antibodies at 4°C overnight and then probed with HRP-conjugated secondary antibodies for 1 hour at RT. Immunoreactive bands were detected by enhanced chemiluminescence with Clarity™ Western ECL Substrate (Bio-Rad; #170-5060) The intensities of the signals were quantified by densitometry using Image J software according to the manufacturer's instructions.

### 2.9. Cell Viability Test

Cell viability was determined by MTT assay. The decidual cells were seeded in 96-well plates at 1×10^5^ cells/ml and cultured for 48h. After being treated with the alcohol extraction of TFCC at various concentrations of 100*μ*g/ml, 10 *μ*g/ml, 1 *μ*g/ml, 0.1 *μ*g/ml, cells were cultured for 24h. The control group was treated with seeding media (0.1%DMSO in DMEM/F12) (5 wells in each group, 100 *μ*l each well).

Cells were incubated at 37°C in 5 mg/ml MTT solution for 3.5 h. After the removal of the MTT solution, 100 *μ*l of dimethyl sulfoxide were added, and the absorbance at the wavelength of 490 nm was recorded with a microtiter-plate reader. Cell viability was normalized with the control culture. The experiment was repeated three times and presented under the form of mean.

### 2.10. Statistical Analysis

Statistical analyses were performed with Student's* t*-test and one-way ANOVA using SPSS 13.0 (SPSS Inc., Chicago, IL) and presented in the form of mean ± standard. p<0.05 was considered statistically significant.

## 3. Results

### 3.1. TFCC Reduces Abortion Rate in Mifepristone-Induced Rat Model

**Figure 1:**
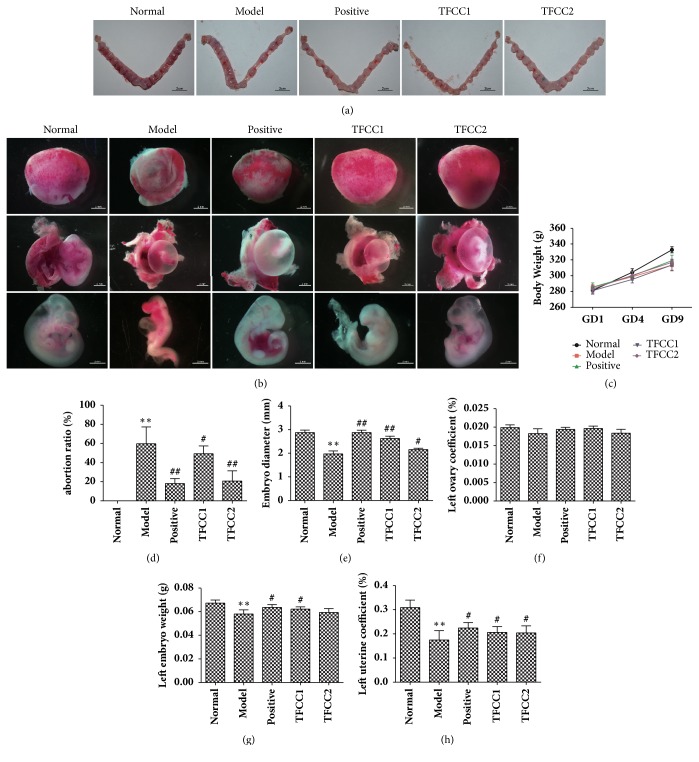
**TFCC reduce abortion rate in model rats**. (a) Anatomy of uterus. Scale bars, 2 cm; (b) the third embryo from left side uterus in each group was observed under stereomicroscope. Scale bar, 2 mm; (c) body weight curve; (d) abortion ratio (number  of  aborted  embryos/number  of  embryos); (e) diameters of embryos; (f) left ovary coefficient ((left  ovary  weight/weight) × 100%); (g) weight of left embryos; (h) left uterus coefficient ((left  uterus  weight/weight) × 100%). Student's* t*-test was applied in the comparison between two groups. “*∗*”* p<*0.05, “*∗∗*”* p<*0.01, “*∗∗∗*”* p<*0.001 compared to the Nor group; “#”* p<*0.05, “##”* p<*0.01 compared to the Mod group.

In order to better assess the anti-abortion effect of TFCC to abortion rat model, we first examined model rat's uterus by visual inspection. The affected uterus was in bamboo-like shape covered with pale white membranous tissue along with internal blood stasis (Figure 1(a)).

Compared to the Nor group and treatment group, both the uterine volume and the number of embryos were significant lower in Mod group. Apart from a small amount of blood stasis observed in TFCC1 group, there were no significant differences between Pos group, TFCC1 group, and TFCC2 group. The third embryo from the left-side uterus of each rat was observed under stereomicroscope (OLYMPUS Corporation, Japan) (Figure 1(b)). Embryo morphology was shriveled in Mod group, which showed that the surface folds were significantly less than the other groups. The trace of absorbed blastocysts along with nondistinctive vascular proliferation could be observed in the Mod group. Embryo morphology and vascular proliferation in TFCC1 and TFCC2 were significantly higher than Mod group.

The rats in Mod showed slower locomotion, depression, and loss of luster in hair compared to other groups. No significant differences were found between the 4th day and 9th day of weight growth (*p>*0.05) among all groups (Figure 1(c)). Comparing with the Mod group, results showed that TFCC can substantially reduce the abortion rate in rats (*p<*0.05 for TFCC1;* p<*0.01 for TFCC2) (Figure 1(d)) and increase the diameter of embryos (*p<*0.05 for TFCC2;* p<*0.01 for TFCC1) (Figure 1(e)) and left uterus coefficient (*p<*0.05) (Figure 1(f)). The weight of left embryos was elevated in TFCC1 (*p>*0.05) (Figure 1(g)). Significant differences in left uterus coefficient could be observed among all groups (*p<*0.05) (Figure 1(h))

### 3.2. TFCC Reduces Decidual Cell Damage and Promotes Serum Gonadal Hormone Level and mRNA Receptor Expression

**Figure 2:**
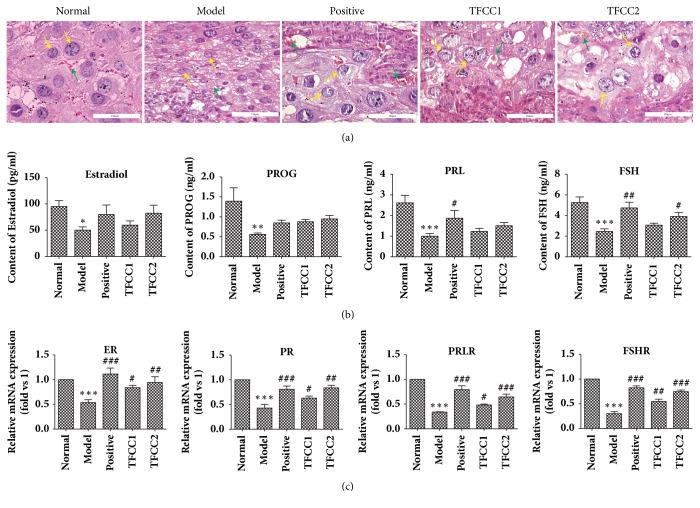
**TFCC increase serum gonadal hormone level and related mRNA receptor expression**. (a) Lesions in decidua tissues. Yellow arrow for decidua cell; green arrow for blood vessels and red blood cells. Scale bars, 150 *μ*m; (b) serum gonadal hormone level quantified by ELISA. (c) Expression of mRNA receptors (ER, PR, PRLR, FSHR) in rat decidua tissue. Student's* t*-test was applied in the comparison between two groups. “*∗*”* p<*0.05, “*∗∗*”* p<*0.01, “*∗∗∗*”* p<*0.001 compared to the Nor group; “#”* p<*0.05, “##”* p<*0.01 compared to the Mod group. Data were representative of** 3** experiments.

To further study the pathological change in uterus after TFCC treatment, the tissues were processed for H&E staining (Figure 2(a)) before being observed under light microscope. Results showed that, compared to Mod group, various types of lesions including edema and degeneration of decidua cell, separation between gland and interstitial tissue, enlarging sponge gland, and local fibrinoid necrosis were observed and the severity was significantly alleviated in Pos group and TFFC1 and TFFC2 group.

Next, we investigated the serum gonadal hormone level in test animals (Figure 2(b)). It was shown that serum gonadal hormone in Mod group was substantially lower than that in Nor group (*p<*0.05 for Estradiol,* p<*0.01 for PROG,* p<*0.001 for PRL and Fsh). Administration of either dydrogesterone or TFCC promotes the secretion of serum gonadal hormone, particularly FSH in TFFC2 group (*p<*0.05). Total RNA was extracted from the uterine decidua tissue and gene expressions were quantified by quantitative RT-PCR. It was demonstrated that mRNA expressions of ER, PR, PRLR, and FSHR were significantly lower in Nor group (*p<*0.001). However, administration of low dose TFCC further increased the expression of ER (*p<*0.05), PR (*p<*0.05), PRLR (*p<*0.05), and FSHR (*p<*0.001); the high dose TFCC has better reversal effect (Figure 2(c)).

### 3.3. TFCC Enhance the Expression of Gonadal Hormone Receptor Protein

**Figure 3:**
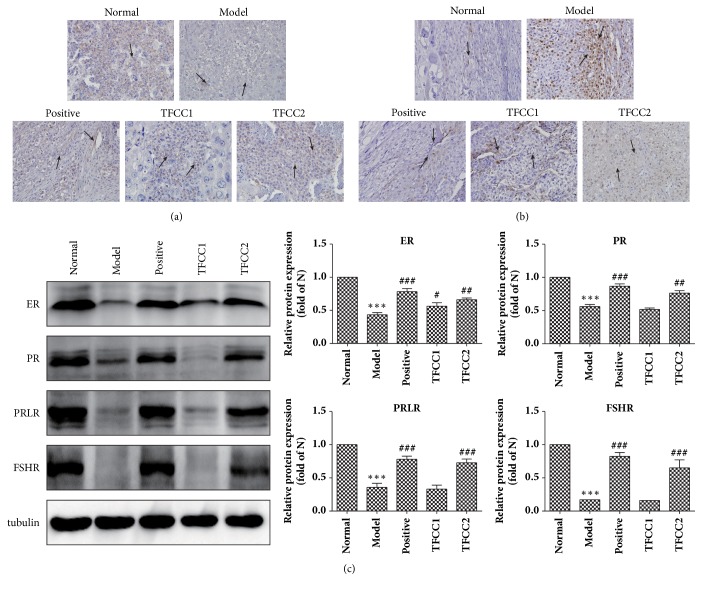
**TFCC enhance the expression of gonadal hormone receptor protein. **Immunohistochemical analysis: (a) protein expression of PR in decidua tissue (solid black arrows); (b) protein expression of p-ERK in decidua tissue (solid black arrows); (c) protein expression of ER, PR, PRLR in Mod group by Western Blot (*p<*0.001). T-test was applied in the comparison between two groups. “*∗*”* p<*0.05, “*∗∗*”* p<*0.01, “*∗∗∗*”* p<*0.001 compared to the Nor group; “#”* p<*0.05, “##”* p<*0.01 compared to the Mod group.

**Figure 4:**
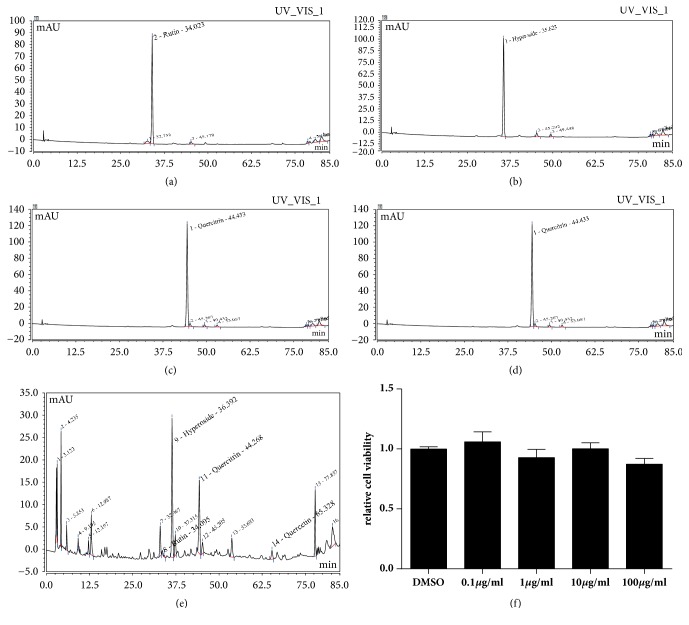
**The components of TFCC effect on cell viability. **(a) rutin (standard sample); (b) hyperoside (standard sample); (c) quercitrin (standard sample); (d) quercetin (standard sample); (e) the image of main components of TFCC identified by HPLC method; (f) MTT results indicated that 0.1*μ*g/ml, 1 *μ*g/ml, 10 *μ*g/ml, and 100 *μ*g/ml concentration of TFCC had no distinctly cytotoxic effect on decidua primary cells. Data were representative of** 3** experiments.

To trace differentiated protein expression related to gonadal hormone receptor, decidua cells were harvested from test animals and fixed with 4% neural paraformaldehyde. Protein expression in decidua cells were examined by Immunocytochemistry methods. Under stereomicroscope observation, positive staining of PR and p-ERK was observed in the nucleus of decidua stromal cells. The expression of both PR (Figure 3(a)) and p-ERK (Figure 3(b)) in decidua tissue reduced significantly in Mol group compared to Nor group (*p<*0.05). When either mifepristone or TFCC were administered to the animals, the protein expressions were rescued and returned to higher level. The trend and degree of change in the protein expression are comparable between TFCC and mifepristone groups.

We further confirmed the observation from ICC by performing Western-blot on total protein extracted from decidua cells. Analysis showed that the protein expression of ER, PR, PRLR, and FSHR in Mod group was significant lower than in other treatment groups (*p<*0.01). The expression of ER had been already upregulated in TFCC1 (*p<*0.05), except PR, PRLR, and FSHR. But the high dose TFCC (TFCC2) could upregulated significantly the expression of PR (*p<*0.01), PRLR (*p<*0.001), and FSHR (*p<*0.001) (Figure 3(c)).

### 3.4. TFCC Has Little Cytotoxicity on Decidua Cells under 100 *μ*g/ml

To better access the potential toxicity that TFCC may have on decidua cells, we first employed HPLC to identify the active components in TFCC. HPLC analysis revealed the major components in TFCC including rutin, hyperoside, quercitrin, and quercetin. Next, we cultured primary decidua cells until third generation and then treated the cells with different concentrations of TFCC (100*μ*g/ml, 10*μ*g/ml, 1*μ*g/ml, 0.1*μ*g/ml) for 24h. Cell viability was determined by MTT assay. At different concentrations of 0.1 *μ*g /ml, 1 *μ*g/ml, 10 *μ*g/ml, and 100 *μ*g/ml of TFCC, no statistical significance was detected in cell viability on decidua cells.

### 3.5. TFCC Downregulates p-ERK and p-38 Expression in Primary SA Decidua Cells

**Figure 5:**
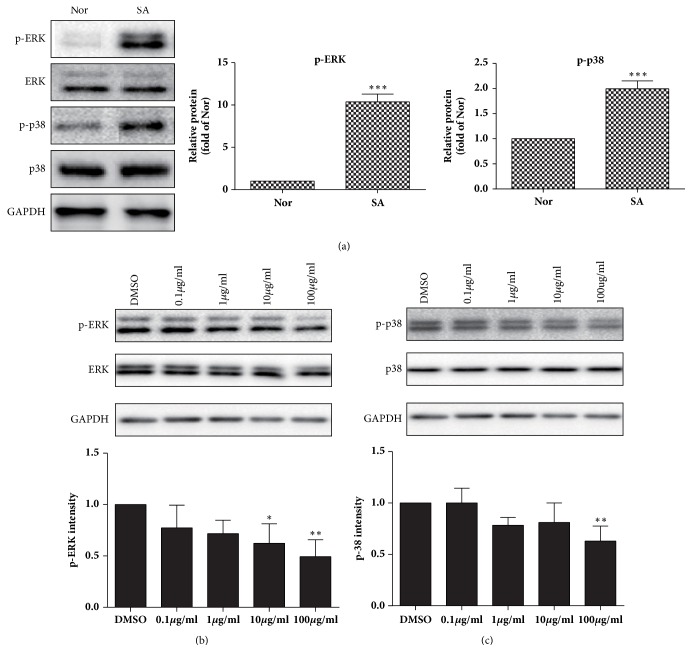
**TFCC downregulate p-ERK and p-38 expression in decidua primary cells. **(a) Phosphorylation of ERK and p-38 from normal early pregnancy and spontaneous abortion (*p<* 0.001); (b) phosphorylation of ERK and p-38 after TFCC treatment in primary decidua cells. *∗P <*0.05, *∗∗P <*0.01 vs DMSO. Data were representative of** 3** experiments.

To demonstrate clinical relevance of TFCC in human patients, we collected human decidua cells from patients with spontaneous abortion and those who willingly terminated pregnancy at first trimester. Western blot results showed that phosphorylation of ERK and p-38 were elevated in decidua cells collected from early pregnancy (*p<*0.001), confirming the data from previous animal models where phosphorylated ERK and p-38 were enhanced on decidua tissue. After administration of TFCC, the phosphorylation level of ERK in decidua cells decreased in a dose-dependent manner. The reduction was most evident at 10 *μ*g/ml concentration of TFCC (*p<*0.05) (Figure 5(b)). A similar trend was observed in p-38. When TFCC concentration was elevated, the level of p-38 decreased. The reduction was most significant at 100 *μ*g/ml of TFCC (*p<*0.01) (Figure 5(c)).

### 3.6. TFCC Upregulate the Expression of Reproduction Associated Receptor

**Figure 6:**
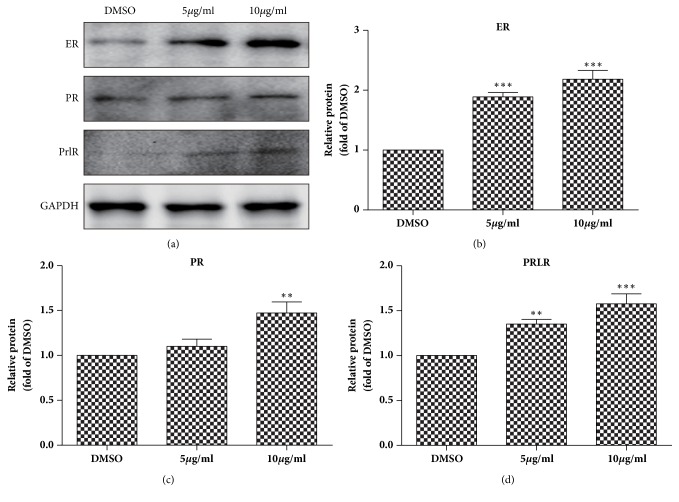
**TFCC upregulate the expression of reproduction associated receptor. **(a) Expression of ER, PR, and PRLR in decidua cells regulated by TFCC; (b) both 5 *μ*g/ml TFCC and 10 *μ*g/ml TFCC could up-regulate the expression of ER; (c) 10*μ*g/ml TFCC could upregulate the expression of PR; (d) both 5 *μ*g/ml TFCC and 10 *μ*g/ml TFCC could upregulate the expression of PRLR. “*∗*”* p<*0.05, “*∗∗*”* p<*0.01, “*∗∗∗*”* p<*0.001 vs DMSO. Data were representative of** 3 **experiments.

To investigate the effect of TFCC on reproduction hormone receptor, we extracted protein from primary decidua cells after 8 hours of TFCC administration and examined the expression of ER, PR, PRLR by Western Blot. Results showed that protein levels of all three hormone receptors were increased significantly by TFCC in a dose-dependent manner (Figure 6).

## 4. Discussion

Spontaneous abortion is a common reproductive disease caused by the dysfunction of decidua cells in maternal fetal interface. It affects approximately 15%-25% of gravida and can lead to severe psychological stress in patients. In this study, we revealed the potential therapeutic effect of TFCC on preventing abortion in DSCs and found that TFCC exerts its anti-abortion effect through interfering MAPK signaling pathway in DSCs. Currently, the main treatment of abortion is still the hormone replacement therapy and immunotherapy. Moreover, the safety of hormone usage during pregnancy remains controversial and both the efficacy and long-term safety of immunotherapy remain unclear. On the other hand, TCM has been employed to treat abortion for thousands of years. In spite of the lack of quantitative evidences, many components have been shown to exhibit clinically effective components in a variety of diseases with unique and advantages [1, 23–25].

In order to better study the pharmacodynamics of TFCC, mifepristone (RU486)-induced abortion rat model is introduced in this study. RU486, a derivative of the norethindrone, was discovered by French researchers in 1982. Because of the similarity in structure between RU486 and progesterone, it is highly effective to compete with progesterone for progesterone receptor binding and thereby causes degeneration and necrosis of decidua and villus tissue, leading to subsequent embryonic death. Thus, RU486 is approved for medical abortion usage by the U.S. Food and Drug Administration in 2000. A recent study indicated that even, at low concentration (0.5*μ*M)* in vitro*, mifepristone may be a contraceptive agent given its inhibitory effect on embryo implantation process during the receptive period [26]. In this study, we observed a universal decrease of not only the level of serum sex hormones (E_2_, P, PRL, FSH) but also the expression of ER, PR, PRLR, FSHR in Mod group decidua tissue compared with Nor group. The decrease in reproductive hormones level and their respective receptors are accompanied by higher abortion rate in Mod group. In summary, mifepristone serves as a successful* in vivo* model for abortion study with physiological relevance.

C. chinensis is an herb in TCM well known for its effect against reproductive system diseases, especially the abortion caused by kidney deficiency. Despite of the fact that C. chinensis has been applied clinically by TCM practitioner, little has been studied on its biochemical composition as well as the molecular mechanism underlying its anti-abortion effect. Without evidence from systematic and quantitative study, it becomes difficult for C. chinensis to benefit larger patient population. One of the best studied components in C. chinensis is TFCC. Previous study using hydrocortisone- induced abortion model in rat showed that TFCC can reverse the expression of testosterone and androgen receptor [27]. Another study showed that TFCC promotes ER expression in the hippocampus, hypothalamus and pituitaries of psychologically stressed rats as well as LHR expression in the ovaries [9], suggesting a potential role of TFCC in regulation reproductive endocrine function. Hormonal imbalance is one of the leading causes of abortion and bringing back the balance of hormone and respective receptors can help to prevent spontaneous abortion. A pilot study using bromocriptine-induced abortion model in rat already showed [13] that TFCC significantly reduced the abortion rate by improving the blood supply in placenta, promoting the expression of PR in decidua and increasing the level of P as well as Prl. However, the exact role of MAPK pathway in the prevention and treatment of abortion of TFCC remained unexplored. In our study, we show that TFCC not only improves embryo quality and decreases the abortion rate in mifepristone-treated rats, but also reverses the expression of hormone levels in model rats in a dose-dependent manner. Meanwhile, TFCC promotes the expression of PR and PRLR to reduce decidua damage caused by competitive binding with PR by mifepristone. This compensatory mechanism functions as the key of TFCC to prevent and treat abortion. Furthermore, we found the expression of p-ERK is higher in Mod group than Nor group. This finding resonates with the result where the expression of p-ERK and p-p38 are elevated in primary decidua cells from spontaneous abortion than that from normal pregnancy. Administration of TFCC* in vivo* and* in vitro* reversed not only the pathological state but also dampened the protein expression of p-ERK and p-38, indicating that MAPK signaling pathway plays a central role in spontaneous abortion and TFCC can effectively intervene with this pathway.

Abnormity in the MAPK signaling pathway has been related to multiple diseases including cancer [28], aortic valve disease [27] and endocrine diseases [29]. In the reproductive system, the MAPK pathway also plays an important role in maintenance of normal pregnancy at the maternal fetal interface. One of the examples is the regulation of growth and differentiation of placenta by ERK / MAPK pathway [30]. Several studies also showed that ERK1/2, JNK, and AKT pathway can promote the proliferation and invasion of DSCs [19, 20]. However, in our study, both animal models and primary decidua stromal cells showed concurrent upregulation of p-ERK and p-p38 with abortion, suggesting their potential roles in spontaneous abortion. In recent study indicated that hyperoside, one of the major components in TFCC, inhibited the phosphorylation of p65/NF-*κ*B, MAPK (including p38, JNK and ERK1/2) in mice with high-carbohydrate/high-fat diet and alloxan-induced diabetes [31]. However, hyperoside had also been reported to significantly increase the protein phosphorylation of p38 mitogen-activated protein kinase (MAPK) and c-Jun N-terminal kinase (JNK) in A549 human non-small-cell lung cancer cells [32]. Therefore, MAPK may serve dual roles in regulating MAPK signaling pathway in TFCC for abortion prevention. In this study, our result demonstrated that TFCC downregulate p-ERK and p-p38 in a dose-dependent manner with a concurrent reduction in abortion rate, indicating that TFCC reverses the pathological process of decidua to suppress abortion by inhibiting MAPK signaling pathway.

## 5. Conclusion

Our study proved that TFCC administered in vivo effectively reduced mifepristone-induced abortion and also had little cytotoxicity effect on human primary decidua cells. Identifying MAPK signaling pathway as a target of TFCC allows future development of therapies for preventing spontaneous abortion.

## Figures and Tables

**Table 1:** The sequence of different primer.

**Primer sequence (5'--3')**	**Primer name**	**Gene Bank**
CCAGCAGGGTGGCTCATC	ER (rat)-F	NM_012689.1
CTGGTGCAACAAGGCCATTC	ER (rat)-R	
GACAACACAAAGCCCGACAC	PgR (rat)-F	NM_022847.1
CGGAAACCTGGCAGAGACTT	PgR (rat)-R	
ACTGGCTGTGTCATTGCTCT	FSHR (rat)-F	NM_199237.1
AAACCTCAGTTCAATGGCGTTC	FSHR (rat)-R	
TTACACGGGGCTCAGGAAAC	PRLR (rat)-F	NM_001034111.1
TTCAGGTTGGCCCCTTCTTC	PRLR (rat)-R	
CCCGCGAGTACAACCTTCTTG	*β*-actin (rat)-F	NM_031144.3
GTCATCCATGGCGAACTGGTG	*β*-actin (rat)-R	

## Data Availability

The data and materials are available from the corresponding author on reasonable request.

## Abbreviations

DSCs: Decidua stromal cells
ER: Estrogen receptor
FSHR: Follicle-stimulating hormone receptor
HPLC: High performance liquid chromatography
MCP3: Monocyte Chemotactic Protein-3
PR: Progesterone receptor
PRLR: Prolactin receptor
RT: Room temperature
RU486: Mifepristone
TCM: Traditional Chinese medicine
TFCC: Total flavones extracted from Cuscuta chinensis.
